# Evaluation of smart TV user interfaces for older adults based on the perception assessment method of analytic hierarchy process (AHP)-fuzzy theory

**DOI:** 10.3389/fpsyg.2026.1848479

**Published:** 2026-08-10

**Authors:** Ting Zhang, Rosalam Che Me, Hassan Alli

**Affiliations:** 1 School of Fine Arts, School of Design, Zhaoqing University, Zhaoqing, Guangdong, China; 2 Department of Industrial Design, Faculty of Design and Architecture, Universiti Putra Malaysia, Serdang, Malaysia

**Keywords:** age-friendly design, analytic hierarchy process (AHP), human-computer interaction, Kansei engineering (KE), older adults in China, smart TVs, usability evaluation

## Abstract

With the deepening of population aging in China, smart TVs have become the most frequently used home digital device for older adults, serving as their core channel for daily information access and entertainment. However, the mismatch between older adults’ perceptual needs, declining sensory and cognitive abilities, and the complex interface design of smart TVs has severely exacerbated the digital divide among this group. Existing studies mostly focus on qualitative age-appropriate design strategies, and lack a targeted, quantitative usability evaluation system that integrates older adults’ subjective perceptual needs and interface performance assessment. This research develops a multi-level usability evaluation index system for older adults’ smart TV interfaces by integrating Kansei Engineering (KE) and Analytic Hierarchy Process (AHP)-fuzzy theory. The system includes 2 first-level, 5 s-level, and 25 third-level indicators. Taking Huawei (Harmony OS) and Skyworth (Android-based) smart TVs as cases, this research conducted a standardized usability test with 15 older adults aged 60–74 in a novice user scenario to eliminate the interference of prior learning experience. The evaluation system’s robustness was preliminarily verified through model sensitivity analysis, with overall score fluctuations below 1.71% under ±10% perturbations of core indicator weights. The results show that within the user experience dimension, interactive usability (local weight: 0.4126) ranks highest in importance, followed by feedback usability (0.3275), which is identified as a high-priority optimization direction due to its low actual scores. Skyworth scored 83.11 in overall usability, while Huawei scored 81.95; both brands scored relatively low in user experience usability (around 79 points), with error prevention, recoverability, and generic function being major issues. The system developed in this study can effectively assess interface usability, providing data support and targeted design guidance for the age-friendly optimisation of smart TV brands. This study has two main limitations: the sample is concentrated in the Zhaoqing region of Guangdong Province, and only two mainstream smart TV systems are evaluated. Future research will broaden the sample size to ensure the universality of the conclusions.

## 1 Introduction

According to the United Nations (1956), a country is considered an ageing society when the proportion of people aged 60 and more exceeds 10% of its total population. According to the Department of Statistics China (2022) National Bulletin on Aging Development in China, the population aged 65 and over in China has reached 267 million, accounting for 18.9% of the total population. As the most frequently used home entertainment and information device for older adults’, smart TVs have become a core carrier for this group to access digital life (Baidu, 2022). Supplementary Figure S1 shows the proportion of people aged 65 and over in each province in China. The analysis shows that in ten provinces of the country (excluding Hong Kong, Macau and Taiwan), the proportion of people aged 65 and over exceeds 20% of the population, especially in Sichuan province, Chongqing municipality and some northeastern provinces in China. The aging population is most pronounced in Liaoning province (23.22%). The population aged 65 and over exceeds 200 million, accounting for 14.2%. However, older adults face challenges in using smart TVs due to limited sensory abilities and interfaces mismatched with their needs(Wu and Hu, 2018; Ouyang and Zhou, 2019; Guo et al., 2023). Existing studies on smart TV design for older adults lack usability evaluation tools.

This study aims to fill this gap by developing an evaluation model integrating KE and AHP-fuzzy theory, with three objectives: (1) Construct a usability index system for older adults’ smart TV interfaces; (2) Compare usability of mainstream smart TVs (Huawei, Skyworth); (3) Provide design guidance for brands. The integration of KE and AHP-fuzzy theory directly addresses the following research gaps: KE captures subjective perceptual needs that typical quantitative methods neglect, while AHP-fuzzy theory provides a clear priority-ranking framework that compensates for the lack of prioritisation in qualitative research.

## 2 Literature review

Age-appropriate design of intelligent products can effectively reduce the digital divide faced by older adults’, and smart TV, as the most frequently used home digital device for this group, is the core scenario for aging-friendly digital design (Wang and Wu, 2022). Previous studies have shown that older Chinese adults spend an average of more than 4 h a day watching TV, which is their most trusted source of information and entertainment (Naudé et al., 2024). However, with the intelligent upgrading of TV systems, a large number of usability barriers have emerged: complex navigation logic, non-intuitive interface layout, and mismatched interaction methods have made it difficult for older adults’ to use smart TVs beyond basic live broadcast functions (Guo et al., 2023; Li et al., 2026). Older adults’ benefit from age-appropriate intelligent products, with TVs being their most frequently used appliance (Wang and Wu, 2022). However, usability barriers (e.g., complex navigation, non-intuitive interfaces) persist. Similarly, older Chinese people spend a lot of time in front of the television every day. Television is indeed the most important source of information for older people who rely on and trust media communication (Wang and Wu, 2022).

Nevertheless, older adults’ also need help to use smart TVs mostly to understand the interaction logic or hierarchical relationships of smart TV pages because their sensory abilities such as vision and hearing are limited (Langdon et al., 2025). In addition, younger users, as the primary consumer group, dominate mainstream product design directions, and the functions and interfaces of their smart TVs do not match the characteristics of older adults’ use (Lu et al., 2024; Guo et al., 2023; Zhou et al., 2024). On top of the behavioral and psychological characteristics of older adults’ that affect the usage effect, smart TVs also suffer from issues such as overly tech-oriented aesthetics, complex operation flows, and an abundance of infrequently used functions (Alam et al., 2019; Guo et al., 2023).

Previous researchers have discussed the design process of smart TV for older adults in China. However, there is a lack of research on the usability of smart TVs for older adults. Lei and Tan (2025) investigated the operation of smart TVs for older adults using a logistic approach and concluded that there are some significant problems in the use of smart TVs for older adults, but that it is necessary to analyze the design strategy for smart TVs. Alam et al. (2019) and Wang and Wu (2022) compared the factors influencing older adults’ smart TV usage with the observational experiment method and proposed learning strategies and design suggestions. However, there is no comparison of the prioritization of design considerations.

AHP and other multi-criteria decision-making (MCDM) methods are widely used in product evaluation (Ali and Marra, 2017; Donegan et al., 1992; Wang et al., 2019). Recent research (Elraaid et al., 2024) on construction project management and (Radovanović et al., 2025) demonstrated the effectiveness of AHP in handling subjective and qualitative data in evaluating university professors. Compared with newer methods such as BWM (Best Worst Method) or FUCOM (Complete Consistency Method), AHP provides a more transparent hierarchical structure, making it more suitable for complex systems with multiple indicators (Ekin and Sarul, 2022). However, the Analytic Hierarchy Process has limitations: it relies on subjective expert judgment, which may introduce bias - here this problem is addressed by combining it with fuzzy theory to quantify uncertainty.

A study has constructed a usability evaluation model for elderly intelligent products using AHP to enhance product usability and user experience (Wang and Yilin, 2024). In addition, AHP has also been used to construct design models for intelligent products for older adults, such as the “Hierarchical Design Model for the Application of Humanistic Care Factors” (HFAHD), which combines AHP and ANP to rank and analyse the importance of humanistic care factors in the design of intelligent elderly care products (Zhou et al., 2023). In designing intelligent products for older adults, the application of AHP is not limited to evaluation and optimization, but also involves identifying user needs, prioritizing product functions, and formulating design strategies. For example, the combination of AHP and Quality Function Deployment (QFD) methods is used to design intelligent elderly products to optimize product design and meet user needs (Li et al., 2022; Li and Li, 2024). KE captures users’ subjective perceptions and combines them with AHP in products targeting older adults (Chen et al., 2023) on smartwatches; (Guo et al., 2017; Xia et al., 2021) on health products, but has never been used on smart TVs. This research is the first to integrate these theories into smart TV interfaces.

Although existing research has proven the severe usability hurdles in smart TVs’ aging-friendly interfaces and investigated the use of AHP-fuzzy theory and KE in the evaluation of senior intelligent products, three fundamental research gaps remain that must be addressed immediately. For starters, approaches are not diverse. The majority of existing research on aging-friendly smart TVs uses qualitative approaches, such as interviews and observations, to generate design ideas, with no quantitative or multidimensional usability evaluation index system, which prevents horizontal comparison and prioritisation of interfaces across brands or versions. A tiny number of quantitative studies focus solely on objective variables, such as operational efficiency, while ignoring older adults’ subjective emotional needs.

In addition, theoretical integration is absent. Most existing evaluation studies use only the AHP or KE methods. AHP excels at building a hierarchical indicator system and calculating weights, but relies too heavily on expert judgement; KE can capture users’ subjective perceptual needs, but struggles to prioritise indicators. No research has yet combined the two methodologies to quantify older adults’ subjective perceptions while prioritising design indicators. Furthermore, there are constraints in research contexts. Most existing usability tests involve users with prior experience with the system under test, which cannot exclude the influence of learning effects on test results and thus struggles to reflect the interface’s inherent usability. There is a lack of targeted research on “novice elderly users” who have no prior experience, even though this demographic is where the digital gap is most severe.

To close the aforementioned shortcomings, this study combines KE and AHP-fuzzy theory to provide an evaluation model. KE is used to extract older adults’ basic perceptual needs and convert them into evaluation indicators, while AHP-fuzzy theory is used to calculate indicator weights and perform comprehensive evaluation. This technique not only overcomes the limitations of a single method but also considers both objective interface performance and subjective user perceptions.

## 3 Method

To fill this gap, this research is to develop a multi-level usability evaluation index system for older adults’ smart TV interfaces, comprising two first-level, five second-level, and twenty-five third-level indicators. First, 126 Kansei words were collected from three sources: peer-reviewed literature, e-commerce user reviews, and age-friendly design guidelines. Subsequently, designers with over 5 years of design experience were invited to review the final Kansei words using the KJ method, determine the five-dimensional classification, and calculate the weighting values. At the same time, product samples were identified based on market share to create a perception rating scale. In the third phase, older adults were invited to participate in the behavioral use of smart TVs and fill in the scale to calculate the final perception weighted scores using the hierarchical analysis method. Supplementary Figure S2 shows the research process of this research.

### 3.1 Collection construction of perception assessment tools

First, a total of 126 Kansei words related to interface usability and user experience were collected from three sources: (1) Existing literature on elderly-oriented interface design and Kansei Engineering; (2) User reviews of smart TVs from mainstream e-commerce platforms (focusing on comments from older users or their family members); (3) Interface design guidelines for elderly-oriented digital products.

#### 3.1.1 Adopting KJ methods in extracting Kansei words

The collected Kansei words were then clustered and screened using the Kawakita Jiro (KJ) Method (Kawakita, 1991). The KJ Method is a qualitative research method that uses affinity clustering to organise scattered qualitative data and is commonly utilised in the word extraction stage of KE (Raymond, 1997). This research recruited three experts from various backgrounds to participate in the filtering process. The qualifications of the three experts are as follows: Expert 1 specialises in industrial design and has 5 years of experience in innovative design services. Expert 2 has 7 years of experience in information and communication technologies and industrial design. Expert 3 brings 9 years of experience in aging health product management and industrial design (Supplementary Table S1).

The screening and clustering procedure is broken into 3 steps. During the independent screening stage, three experts excluded duplicate, semantically unclear, and unnecessary terminology to the usability of smart TV interfaces, leaving only 58 vocabulary items pertinent to the senior user experience. The inclusion criteria are: (1) mentioned at least three times in the literature or user reviews; (2) semantic clarity and direct correlation to interface design aspects. The exclusion criteria are: (1) terms with over 80% semantic overlap; (2) Kansei words that describe product hardware performance instead of interface interaction (Supplementary Figure S3). The correspondence between all 25 third-level Kansei indicators and specific usability pain points of older adults is detailed in Supplementary Table S13, linking each perceptual indicator to real usage barriers faced by older adults.

#### 3.1.2 Establishment of the hierarchical structure system

This research develops a four-layer hierarchical evaluation model based on Norman (2024) three-level theory of emotional design (perception layer, behaviour layer, and reflection layer) and the usage characteristics of smart TV interfaces.

The target layer (B) refers to the overall usefulness of the smart TV interface for aging. The level 1 indicator layer (2) includes appearance-perceived usability (Ba) and user experience usability (Bb). The usability of appearance perception corresponds to the Norman theory’s perception layer, reflecting older adults’ first impressions of interface visual elements; the usability of user experience corresponds to the behaviour and reflection layers, reflecting older adults’ operational experience and emotional feelings while using.

The 5 s-level indicators are distributed across two first-level dimensions: under Appearance-perceived Usability (Ba) are Visual Usability (Ba1) and Interface Usability (Ba2); under User Experience Usability (Bb) are Feedback Usability (Bb1), Interactive Usability (Bb2), and Emotional Usability (Bb3). These five aspects are based on the ISO 9241-11 (Dietlein and Bock, 2019) usability standards (effectiveness, efficiency, and satisfaction) and have been enhanced to include the perceptual and cognitive qualities of older persons. Third-level indicator layer (25 Kansei words): Each level 2 indicator has 5 Kansei words retrieved using the KJ approach, with precise related correlations illustrated in Supplementary Figure S4. Five Kansei words were chosen for each fundamental dimension, as is normal practice in Kansei Engineering. This setting ensures that the dimension is fully covered while avoiding indicator redundancy and decreasing the response load for older participants.

#### 3.1.3 Evaluation of the weights of fuzzy assessment indicators

The fuzzy comprehensive evaluation method is grounded in fuzzy set theory. It integrates AHP-derived weights to define the membership function between evaluation factor sets and rating rubric sets. Through linear fuzzy transformation, a multi-indicator problem is aggregated into a single comprehensive score for holistic evaluation. In this paper, the fuzzy assessment method is applied in combination with the scoring value given by the expert assessment method to construct a mathematical model and the evaluation results deriving from the comprehensive consideration of the influence of all factors on usability. The multi-level comprehensive fuzzy assessment method divides all evaluation indicators into several levels. Based on the order from low to high, the indicators at each level are comprehensively evaluated one by one and the evaluation results are obtained.

Weight Determination via Expert Judgment. Based on the determination of the valuation indices, an expert assessment is carried out. Based on the results of the expert assessment, a comparison matrix is created, a hierarchical analysis is carried out and a consistency test is performed to determine the weighting of the indices and thus create the smart TV index system. A total of 8 experienced professionals were invited to conduct the expert assessment and the evaluation method was determined on a scale of 1–9. The 8 experts included 4 senior industrial designers, 2 interaction design researchers, and 2 gerontechnology specialists, all with at least 5 years of relevant experience. The specific process is as follows. This research used the geometric mean method to compute the weight vector of the judgment matrix. The specific stages are as follows.

1. Create a judgement matrix: Using the target layer as the assessment criterion, pairwise comparisons were conducted for indicators at each hierarchical level. Taking the target layer as the criterion, the two first-level indicators were compared pairwise; under each first-level dimension, the corresponding second-level indicators were compared pairwise, and the 1–9 scale method proposed by Saaty (1988) was used for pairwise comparison (Supplementary Table S2). This scale is the most widely used standard in AHP research. The element $b_{\mathrm{ji}}$ of matrix B represents the importance of indicator $B_{i}$ relative to $B_{j}$ , satisfying:
$$b_{\mathrm{ij}}>0,b_{\mathrm{ii}}=1,b_{\mathrm{ji}}=1/b_{\mathrm{ij}}$$
  The values of the judgment matrix type are usually taken on a scale of 1–9, so that the importance weights are quantified numerically, and the following table shows the meaning of importance and the scale values (Supplementary Table S2).
  With regard to all usability within the system of intelligent metrics, a comparison matrix is formed according to the results of the expert ratings, which looks as follows (Supplementary Table S3–S11).
2. Calculate the weight vector: Multiply the elements in each row of the judgement matrix, The geometric mean method was used to calculate weight vectors: multiply the elements in each row of the judgment matrix, take the n−throot of the product ( n = number of indicators), and normalize the results to obtain the weight vector w . Using the five three-level indicators from the visual usability dimension as an example, the calculation procedure is as follows:
$$W_{i}=\sqrt[{5}]{\prod_{j=1}^{5}b_{\mathrm{ij}}}\;\;\;(i=1,2,3,4,5)$$
  The geometric mean values of each indicator calculated are: $W_{1}=1.2817$ , $\;W_{2}=1.1352$ , $W_{3}=1.2703$ , $W_{4}=0.7473$ , $W_{5}=0.8048$ . Normalized to obtain weight vector:
$$w=[\begin{array}{c} 0.2552\\ 0.2222\\ 0.2409\\ 0.1352\\ 0.1465 \end{array}]$$
  The above results show that the weights of the five tertiary indicators under visual usability are: Concise (25.52%), Regular (22.22%), Fashionable (24.09%), Attractive (13.52%), and Aesthetic (14.66%), which are fully consistent with the weight results in Supplementary Table S13.
3. Calculate the maximum eigenvalue:
$$\lambda_{\max}=\sum_{i=1}^{n}\frac{{\left(\mathrm{Aw}\right)}_{i}}{nw_{i}}$$
  Where Aw is the product of the judgment matrix and the weight vector. Calculate the maximum eigenvalue of the visual usability dimension.
$$\lambda_{\max}=5.2850$$
4. Consistency check: Calculate the consistency index CI and consistency ratio CR :
$$\mathrm{CI}=\frac{\lambda_{\max}-n}{n-1},\mathrm{CR}=\frac{\mathrm{CI}}{\mathrm{RI}}$$
  Among them, RI is the random consistency index (Supplementary Table S12). When CR≤0.1 , it is considered that the judgment matrix has satisfactory consistency.

The dimensions of visual usability, with CI =0.0713, RI =1.12, and CR =0.0636 ≤ 0.1, passed the consistency test. The calculation process for visual usability is presented in the main text as an example. Complete judgment matrices, *λ*_max_, CI , RI , and CR values for all levels are provided in Supplementary Table S4–S11. All judgment matrices meet the consistency requirement, with CR values below 0.1. The presentation of AHP calculation procedures in this study follows the standard format widely used in similar design evaluation research (Wang et al., 2025; Wang et al., 2026).

### 3.2 Fuzzy hierarchical analysis

This stage is based on hierarchical analysis, which weights the index dimensions derived in the first stage and combines them with product samples and users to evaluate usability. It uses hierarchical fuzzy analysis to derive the final model for evaluating the usability of smart TVs.

#### 3.2.1 Confirmation of product and user samples

This research selects Huawei (HarmonyOS) and Skyworth (customised on Android) smart TVs as research subjects. Two brands were chosen based on three factors. Android systems account for more than 80% of China’s smart TV market, and Skyworth is a major Android brand with a specialised senior mode. Huawei HarmonyOS is a self-developed operating system with a distinct icon-based design. Together, they cover the most common system types in China (Supplementary Figure S5). However, the two brands use distinct age-friendly design techniques, which can fully reflect the consequences of different design ideas. Both are well-known domestic brands, which reduces the impact of brand unfamiliarity on test outcomes (Supplementary Table S14).

Furthermore, Huawei’s smart TVs prioritise visual interface optimisation, employing large, high-contrast icon designs; Skyworth Smart TV has created the “Elderly Mode,” which simplifies the user experience and comes equipped with a large-button remote control. The design changes between the two can properly capture the effects of various aging design concepts. Both brands are well-known domestic brands with strong recognition among older adults’, reducing the impact of brand unfamiliarity on test outcomes.

The sample sizes in existing sensory engineering studies vary significantly, ranging from 9 to 159 participants, with about 30% of studies including 24 or fewer participants (Almagro, 2011). Nielsen claimed that 15 users could uncover more than 99% of interface usability flaws during exploratory usability testing, a finding that has been frequently implemented in human-computer interaction user research. Instead of conducting statistical inference on a large population, the primary goal of this study is to uncover aging usability difficulties with smart TV interfaces and build an exploratory evaluation model. As a result, a sample size of 15 people is appropriate. This sample size is primarily suitable for exploratory model construction and usability problem identification. Statistical power for population-level inference is limited, and subsequent large-sample studies can further validate the model.

This study enrolled 15 community-dwelling older adults in Zhaoqing City who met the following criteria: (1) they were between the ages of 60 and 74; (2) they had normal vision or corrected vision and normal hearing; (3) they were capable of independently completing basic smart TV operations; and (4) they had no experience using Huawei HarmonyOS or Skyworth’s customised Android system, ensuring testing was conducted in novice user scenarios. Supplementary Table S16 shows the participants’ demographic characteristics.

#### 3.2.2 Task setting

There are a variety of voice and gesture controls for smart TV systems on the market today, and the remote control is still the main interaction device for smart TVs in China. Smart TVs are categorised into live streaming systems and additional systems. They are usually equipped with two remote controls, one for controlling live streaming and the other for controlling other system interfaces. This study focuses on the interaction of standalone system interfaces to understand why functions other than live streaming are underutilized. In this study, the smart TV task flow of (Dou et al., 2019) was cited, combined with the perceptual vocabulary with some deletions and adjustments. Supplementary Table S15 shows eight standardised tests meant to cover common usage scenarios of older individuals. All tasks were performed using only the remote control, except for voice and gesture actions.

## 4 Results and analysis

### 4.1 Results

Supplementary Table S16 presents the demographic characteristics of the 15 participants. All met the novice user inclusion criteria and were residing in Zhaoqing, Guangdong Province, ensuring the reliability of the test results.

Most of the invited older users were retired (86%), two of them were rehired to continue working in their position at the school after retirement, and all of them had a retirement salary or allowance from their children. The researchers found that most of the older adults with a high school diploma or higher lived with a partner and did not live with their children, even in the same city. Fifteen of the older users had experience with smart products and smart TVs.

Evaluation factor sets and rubric sets were established based on the indicator system. Based on the above five rubrics and the corresponding 25 third-level indicators that make up the rubrics, a comprehensive multi-level fuzzy assessment of the usability of smart TVs is conducted. The first-level fuzzy assessment indicator is set as $U_{i}$ . The set of specific value-added factor rubrics for the rater is created as a five-level rubric set v = {v1, v2, v3, v4, v5} = {1, 2, 3, 4, 5}. The first level fuzzy assessment set can be expressed as $U_{i}$ = W × R , where W is the weight vector of the corresponding level and R is the membership matrix, where W denotes the feature vector. Similarly, by combining the five second-level indicators in the evaluation system of this paper, the fuzzy assessment indices of the second level are determined, which can be expressed as follows:

$$U_{\mathrm{ij}}=(W_{1},,,,W_{2},,,,W_{3},,,,W_{4},,,,W_{5})\times [\begin{array}{c} A1\\ A2\\ A3\\ A4\\ A5 \end{array}]$$

Based on the evaluation of fifteen older adults invited to this study on the three-level indicators for the evaluation of smart TVs, the results of the rubric set were summarised in relation to the five-level evaluation of five-point rating scale (1 = strongly disagree, 2 = disagree, 3 = neutral, 4 = agree, 5 = strongly agree).

$$\begin{array}{l} C_{B}=W_{B}\times R_{B}=(0.4000\;\;0.6000)\\ \times (\begin{array}{ccccc} 0.0734 & 0.2335 & 0.1457 & 0.1957 & 0.3516\\ 0.1167 & 0.3482 & 0.1528 & 0.2036 & 0.1788 \end{array})\\ =(0.0994\;0.3023\;0.1500\;0.2004\;0.2479) \end{array}$$

The last two smart TVs therefore have an overall rating of Huawei (81.95) and Skyworth (83.11). The ratings for Level 1, Level 2 and Level 3 are shown below (Supplementary Figure S6–S8). Supplementary Figure S6 shows the first level indicators for the usability assessment metrics, appearance and user experience usability of the two smart TVs. The two brands showed similar overall scores, and both achieved relatively low scores in user experience usability: 79.80 for Huawei and 79.97 for Skyworth.

Supplementary Figure S7 presents the usability scores of the five second-level indicators. Under the appearance-perceived dimension, visual usability scores were 91.45 (Huawei) and 81.81 (Skyworth), and interface usability scores were 82.05 (Huawei) and 90.81 (Skyworth). Under the user experience dimension, feedback usability scores were 70.33 (Huawei) and 73.16 (Skyworth), interactive usability scores were 84.68 (Huawei) and 87.54 (Skyworth), and emotional usability scores were 83.97 (Huawei) and 76.54 (Skyworth). Significant between-brand differences were observed in visual, interface, and emotional usability, while interactive usability showed a smaller score gap.

Supplementary Figure S8 shows the third-level of usability-weighted score indicators, the primary rating scales for older adults. The assessment of the different perceptual terms varies significantly between the two TV brands, with a focus on visual and overall feeling evaluation, with Huawei scoring higher than Skyworth. On the three-level metrics of Generic, Error Prevention, Reported, Learnable and Helpful, both scores were lower, ranging from 65 to 75 points.

The total usability scores for the two brands are 81.95 points for Huawei and 83.11 points for Skyworth. Paired-samples t-test showed no statistically significant difference in overall usability between the two brands (*t*(14) = 1.32, *p* = 0.20), indicating that the two brands’ overall age-friendly usability performance is comparable, with each brand exhibiting advantages in specific dimensions. This non-significant result should be interpreted with caution, as the small sample size may lead to insufficient statistical power and potential Type II error. The brand comparison in this study is exploratory and preliminary.

Paired-samples t-test revealed that Huawei significantly outperforms Skyworth in visual usability (91.45 vs. 81.81, *t*(14) = 4.26, *p* < 0.001). This advantage stems from three core design features: (1) a 24 × 24 dp large-sized icon, 30% larger than the industry standard; (2) a high contrast colour scheme (text to background contrast ≥ 4.5:1); and (3) a flat style icon design, reducing unnecessary decorative elements. This finding is consistent with Mannheim et al. (2019), which discovered that clear and high contrast visual design can considerably increase the first interface comprehension capacity of older adults. Although Huawei outperforms Skyworth in visual usability, its overall score is not higher. This is because user experience usability accounts for 60% of the overall weight, which is higher than appearance-perceived usability (40%). Huawei’s lower scores in feedback, error prevention, and other user experience indicators offset its visual advantage.

Paired-samples t-test confirmed that Skyworth significantly outperforms Huawei in interface usability (90.81 vs. 82.05, *t*(14) = 3.89, *p* < 0.001). This advantage stems from three design optimizations in Skyworth’s elder mode: (1) simplified information architecture that reduces navigation levels; (2) all interactive elements paired with explicit text prompts instead of abstract icons; (3) a dedicated large-button remote control with button spacing ≥ 10 mm to reduce accidental touches. This supports the observation by Ouyang and Zhou (2019) that novice older users rely more on explicit textual assistance than abstract visual signals.

For interactive usability, Skyworth also achieved a higher mean score than Huawei (87.54 vs. 84.68), with a mean difference of 2.86 points. The smaller score gap indicates that the two brands have comparable performance in operation efficiency. Skyworth’s slight advantage mainly comes from shorter task paths for common functions such as screen mirroring.

It is worth noting that both brands have low scores in error prevention (Huawei: 65.33 points; Skyworth: 72.00 points) and recoverability. According to our observations, 4 out of 15 participants (approximately 27%) were unable to quickly return to the previous page after accidentally clicking on an advertisement pop-up, which is closely related to the cognitive characteristics of older adults: as they age, their working memory capacity decreases, their executive control ability weakens, and they find it difficult to remember complex error recovery steps; At the same time, the lack of secondary confirmation for irreversible operations might intensify older adults’ operational anxiety and undermine their trust in the system. This result is consistent with the research conclusion of (Dou et al., 2019) that error management is the core pain point for the use of intelligent products by older adults’.

### 4.2 Model sensitivity analysis

#### 4.2.1 Purpose and objects of analysis

This research constructs a usability evaluation model for older adults using smart TVs based on the Analytic Hierarchy Process (AHP) and fuzzy theory. The core conclusions depend on the stability of indicator weights and user rating data. Model sensitivity analysis aims to examine the fluctuation of evaluation results when key parameters (weights of high-priority indicators, user ratings) are slightly perturbed, thereby verifying the robustness of the model.

Based on data from this research, two types of core parameters are selected as perturbation objects.

Weights of high-priority secondary indicators: According to Supplementary Table S5, within the user experience usability dimension, feedback usability (weight: 0.3275) and interactive usability (0.4126) have the highest weights and significantly impact the overall score, thus being chosen for weight perturbation.

User ratings of low-scoring tertiary indicators: As shown in Supplementary Figure S8, tertiary indicators such as “error prevention,” “recoverability,” and “generic” have low scores (65–75 points) and are directly related to operational barriers for older adults. Their user ratings are selected for perturbation analysis.

#### 4.2.2 Perturbation scheme design

Referring to conventional methods in multi-criteria decision-making model sensitivity analysis, perturbation ranges are set as ±5, ±10% (to simulate reasonable deviations in expert weight judgments) and ±1 point (to simulate errors in user ratings). The specific schemes are as follows (Supplementary Table S17).

#### 4.2.3 Impact of weight perturbation on overall usability scores

After adjusting the weights of feedback usability and interactive usability, the overall usability scores of Huawei (original score: 81.95) and Skyworth (original score: 83.11) changed as follows (Supplementary Table S18).

When the weights of the two high-priority indicators are perturbed within ±10%, the change rates of the overall scores are ≤1.71%, with minimal fluctuations, indicating that the model is insensitive to weight perturbations.

#### 4.2.4 Impact of user rating perturbation on secondary Indicator scores

After perturbing the ratings of “error prevention” and “recoverability” by ±1 point, the scores of the secondary indicator “feedback usability” (Huawei: original 70.33; Skyworth: original 73.16) changed as follows (Supplementary Table S19).

After perturbing the ratings of low-scoring tertiary indicators, the change rates of feedback usability scores are ≤0.81%, with negligible impact on overall results, indicating the model is also robust to user rating perturbations.

#### 4.2.5 Statistical tests

Paired-samples *t*-tests were conducted to verify the significance of score differences before and after perturbations (significance level *α* = 0.05):

There were no significant differences in overall scores before and after weight perturbations for Huawei (*t* = 1.18, *p* = 0.26) and Skyworth (*t* = 1.25, *p* = 0.23).

There were no significant differences in feedback usability scores before and after user rating perturbations (Huawei: *t* = 0.92, *p* = 0.38; Skyworth: *t* = 0.97, *p* = 0.35).

## 5 Discussion

This research developed a tailored usability evaluation system for smart TV interfaces to support evidence-based age-friendly optimization for smart home products.

### 5.1 Theoretical contributions of the evaluation system

This study is the first to integrate KE and AHP-fuzzy theory into the usability evaluation of smart TV interfaces for older adults, which makes two core theoretical contributions. First, the constructed index system fills the gap of lacking a quantitative evaluation tool for smart TV age-appropriate design, which covers both the objective interface performance and the subjective perceptual needs of older adults, making up for the deficiency of existing evaluation systems that only focus on operational efficiency. Second, this study clarifies the weight priority of each usability indicator for older adults, and confirms that interactive usability has the highest weight in the user experience dimension, while feedback usability is the highest-priority optimization direction due to its lowest actual scores, which enriches the theoretical research on age-appropriate interface design for older adults.

### 5.2 Interpretation of core experimental findings

The experimental results show that the overall usability score of Skyworth smart TV (83.11) is slightly higher than that of Huawei (81.95), but the two brands have different advantages in different dimensions. Huawei has a significant advantage in visual usability (91.45 vs. 81.81), which is mainly due to its high-contrast, large-size icon design that effectively reduces the visual processing load of older adults with declining vision; this is consistent with the findings of Mannheim et al. (2019). In contrast, Skyworth performs better in interface usability (90.81 vs. 82.05), which benefits from its clear text prompts and simplified information architecture. This finding confirms that older adults, especially novice users, rely more on explicit textual guidance than on abstract icons in the initial use stage (Mingshun, 2014). Skyworth also shows a slight advantage in interactive usability (87.54 vs. 84.68), mainly due to its shortened operation paths for frequently used functions.

Notably, both brands have low scores in error prevention and recoverability, which is the core pain point for older adults. The observation results show that nearly one third of participants (4 out of 15, 27%) could not quickly return to the previous page after accidentally clicking on an ad pop-up, which is because neither system provides a one-click return function. For novice older users who lack troubleshooting experience, such design defects will directly lead to operation frustration and even give up using the product. Low scores in error prevention and recoverability correlate with age-related cognitive decline. Working memory capacity decreases from 7 ± 2 chunks in young adults to 4 ± 1 chunks in older adults, making multi-step error-recovery processes more difficult to remember. Furthermore, poorer executive control ability causes increased anxiety when confronted with unexpected scenarios such as pop-up adverts, reducing operational confidence and willingness to use the system.

This study found that interactive usability has the highest local weight (0.4126) within the user experience dimension, followed by feedback usability (0.3275). Notably, feedback usability has the lowest scores among all second-level indicators, making it a critical optimization priority with significant theoretical and practical implications. From the standpoint of cognitive psychology, older persons have a higher perception threshold and are less receptive to faint visual and aural information. As a result, unambiguous, multi-channel feedback can assist older persons in determining whether the procedure was effective while reducing uncertainty. Thus, providing visible (button colour change), auditory (prompt sound), and tactile (remote control vibration) feedback after selecting an action can considerably increase older adults’ operational confidence. The existing smart TV interface generally lacks timely and unambiguous feedback, a major source of annoyance for senior users.

### 5.3 Methodological rationality analysis

Although emerging multi-criteria decision-making methods such as BWM and FUCOM can reduce the subjective bias of expert evaluation, this study still chooses AHP-fuzzy theory as the core method, mainly based on two considerations. First, the hierarchical structure of AHP is highly compatible with the multi-level evaluation index system constructed in this study, which can clearly decompose the complex usability evaluation problem into multiple levels of indicators, and realize the transparent weight calculation of each indicator (Donegan et al., 1992). Second, the combination of AHP and fuzzy theory can effectively quantify the subjective perceptual data of older adults collected by KE, which solves the limitation that AHP relies too much on expert judgment, and makes the evaluation results more in line with the real use experience of older adults (Wang and Yilin, 2024). In terms of measurement validity, the evaluation system has good content validity, as it is built on classic theoretical frameworks (Norman, 2024) and reviewed by multidisciplinary experts. The sensitivity analysis verifies the internal robustness (structural validity) of the model. Future studies will further examine criterion validity by comparing the system with established usability scales such as the System Usability Scale (SUS).

### 5.4 Practical implications for age-appropriate design

Based on the weight results of the evaluation system and the experimental findings, this study puts forward three targeted design optimization suggestions for smart TV brands, sorted by priority:

1. First priority: Optimize the error prevention and recoverability of the system. Add a one-click return to the home page function on the remote control and interface, set up a secondary confirmation for irreversible operations, and block non-essential ad pop-ups to reduce the operation risk of older adults.
2. Second priority: Improve the feedback usability of the interface. Add clear visual, auditory, and tactile feedback for all click operations, so that older adults can clearly perceive whether the operation is successful; optimize the voice feedback function to provide friendly operation guidance for older adults.
3. Third priority: Balance the visual design and interaction flow. Learn from Huawei’s high-contrast, large-size icon design to improve the visual clarity of the interface; at the same time, refer to Skyworth’s streamlined task flow to reduce the operation steps of common functions, and match clear text prompts for all interactive elements.

Although this study examined only smart TVs from two brands, Huawei and Skyworth, the findings are generalisable. At the system level, Skyworth’s design features reflect the vast majority of Android-based custom smart TV systems. In contrast, Huawei’s design characteristics point to the future direction of independently built operating systems. As a result, the study’s findings apply to both types of systems.

Furthermore, iconography, navigation, feedback, error management, and other universal design features make up the foundation of the smart TV interface. The design recommendations made in this study are also applicable to other smart home products, such as smart set-top boxes and smart speakers. Although the sample is concentrated in the Zhaoqing area of Guangdong, the core findings regarding the criticality of error prevention and feedback usability are rooted in the universal cognitive aging characteristics of older adults, and sensitivity analysis confirms that the core conclusions are sound. Naturally, older persons may have varied demands depending on their location and degree of digital literacy, which will be investigated in more detail.

## 6 Limitation and generalizability

This study has two main limitations. First, the research samples are all from Zhaoqing City, Guangdong Province (Yang et al., 2023). The digital literacy level of older adults in Guangdong is higher than the national average, which may have a certain impact on the generalizability of the results. However, the sensitivity analysis results show that when the weight of the core indicators is perturbed within ±10%, the fluctuation of the overall score is less than 1.71%, indicating that the core conclusion that “feedback usability, error prevention and recoverability are the core needs of older adults” is robust and has certain universal applicability. Second, this study only selected two smart TV systems as research cases, and the evaluation results may not be fully applicable to other systems such as Tizen.

Future research can be carried out from three aspects: (1) Expand the research sample to cover older adults in different regions of China with different digital literacy levels, to further verify and optimize the evaluation system; (2) Include more smart TV systems and brands in the evaluation, to enrich the research cases; (3) Cross-validate the evaluation model with other multi-criteria decision-making methods such as BWM and FUCOM, to further improve the accuracy and stability of the evaluation system.

## 7 Conclusion

This study integrates Kansei Engineering and AHP-fuzzy theory to construct a multi-level usability evaluation index system for smart TV interfaces targeting older adults in China. At the first level, user experience usability (weight: 0.6000) has higher overall importance than Appearance-perceived Usability (0.4000), indicating that operational experience contributes more to older adults’ overall evaluation of the interface. At the second level, within the appearance-perceived dimension, Interface Usability (local weight: 0.6667) is more important than visual usability (0.3333); within the user experience dimension, Interactive Usability (local weight: 0.4126) ranks first, followed by Feedback Usability (0.3275). At the third level, error prevention, recoverability, and generic function are the lowest-scoring indicators and the highest-priority directions for future age-friendly optimisation. In addition, Huawei and Skyworth have comparable overall age-friendly usability performance, but each has its own advantages. Huawei excels in visual design, while Skyworth excels in optimizing interactive processes. Sensitivity analysis shows that the evaluation model constructed in this research has good robustness and can effectively quantify the age-friendly usability of smart TV interfaces.

The proposed index system effectively reflects older adults’ needs, offering a practical tool for brand optimisation. To enhance generalizability, future research should expand to multi-regional samples in China, include more brands and systems (e.g., Tizen), cross-validate the AHP model with methods like BWM or FUCOM, and focus on error prevention/recovery features tailored to novice older users, given their low scores in these areas. These steps will refine the framework and better support age-appropriate smart TV design.

## Data Availability

The original contributions presented in the study are included in the article/Supplementary material, further inquiries can be directed to the corresponding author/s.
