# Supplementary material for: Evaluation of smart TV user interfaces for older adults based on the perception assessment method of analytic hierarchy process (AHP)-fuzzy theory
Source: Front Psychol. 2026 Aug 10;17:1848479. doi: 10.3389/fpsyg.2026.1848479 (PMC13500268; doi:10.3389/fpsyg.2026.1848479)
Supplement: Supplementary file 1 [file Data_Sheet_1.PDF]

Supplementary Material

1 Supplementary Figures and Tables

1.1 Supplementary Figures

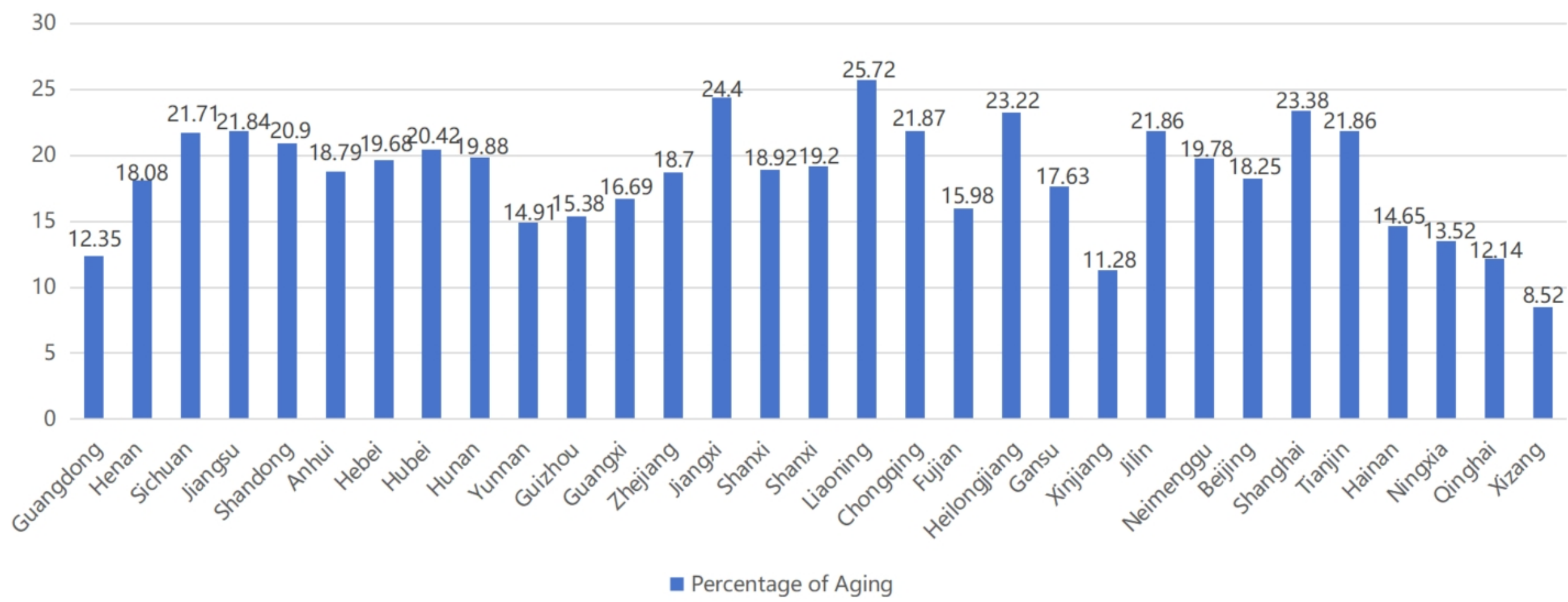

Supplementary Figure 1. Percentage of Aging in China by Province (2022; "Data Visualization, the Degree of Aging in Guangdong Province Is Still Low, Shenzhen, Dongguan, 'the Youngest'" 2022).

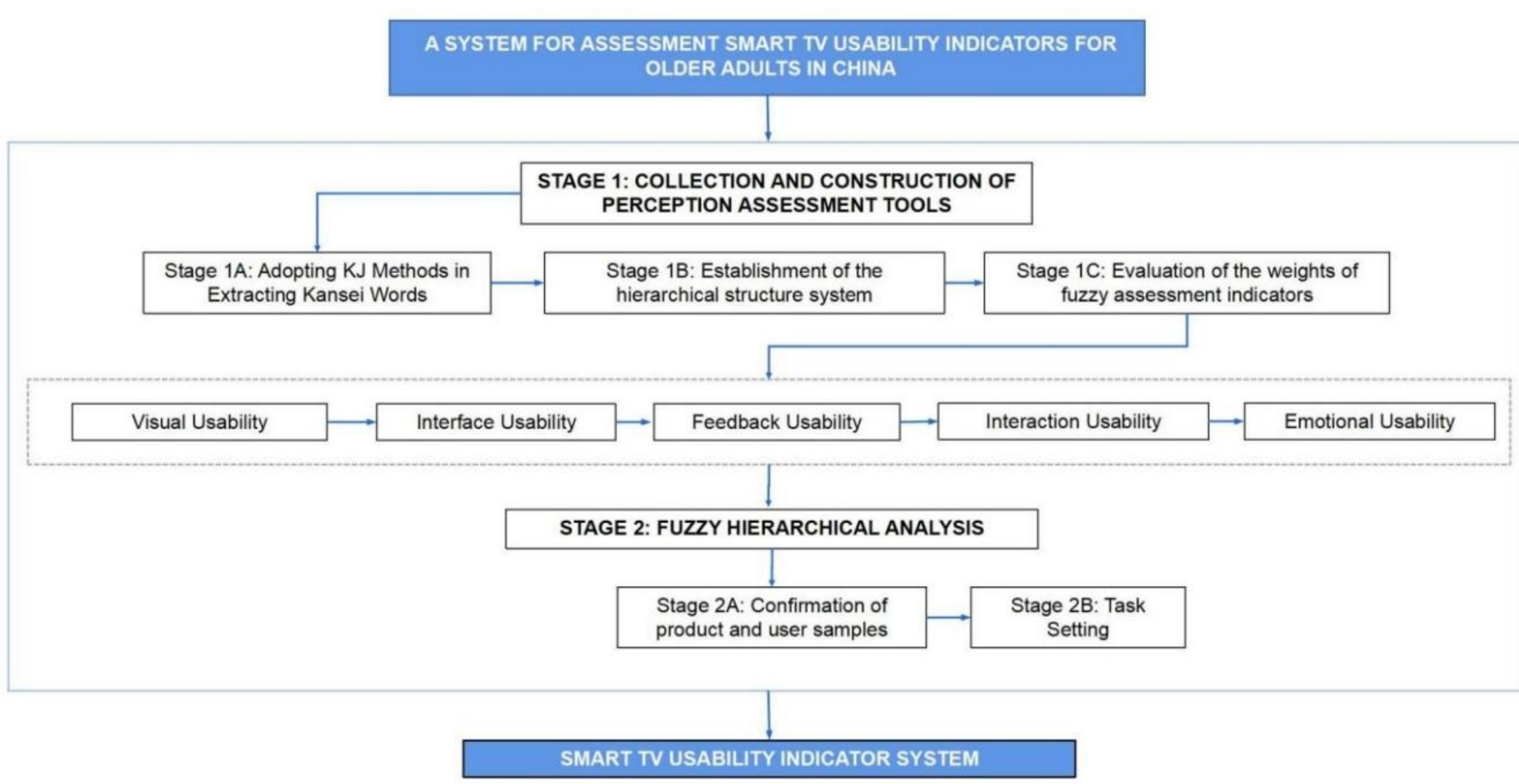

Supplementary Figure 2. Research process

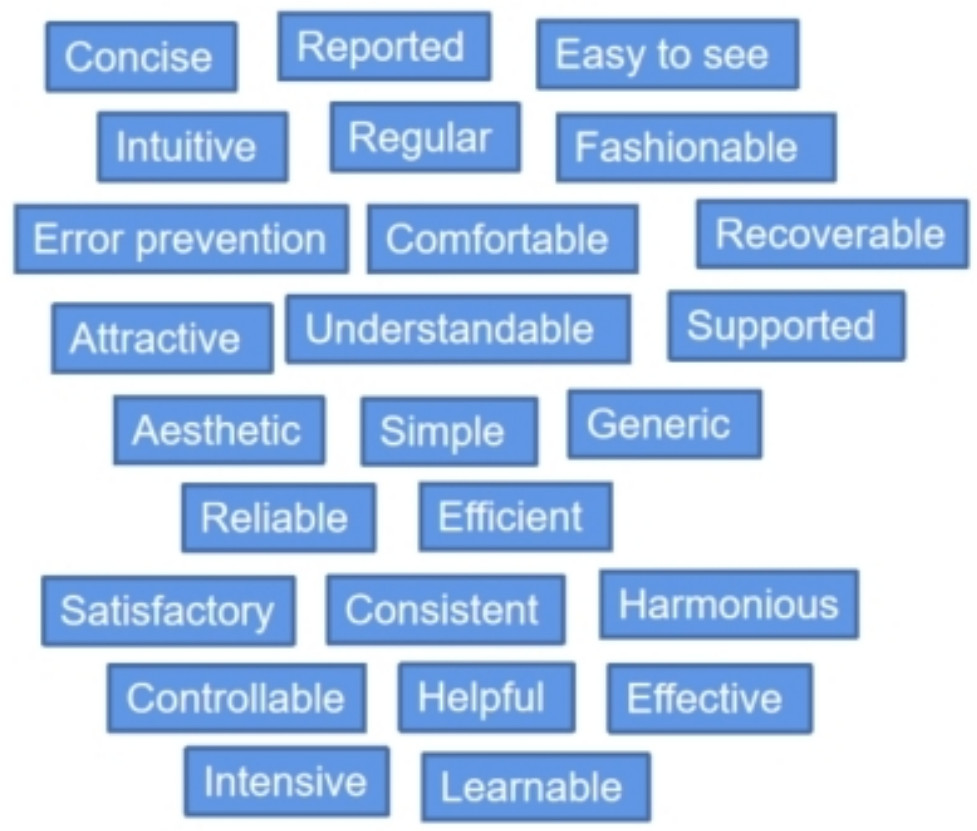

Supplementary Figure 3. Tertiary Kansei Words

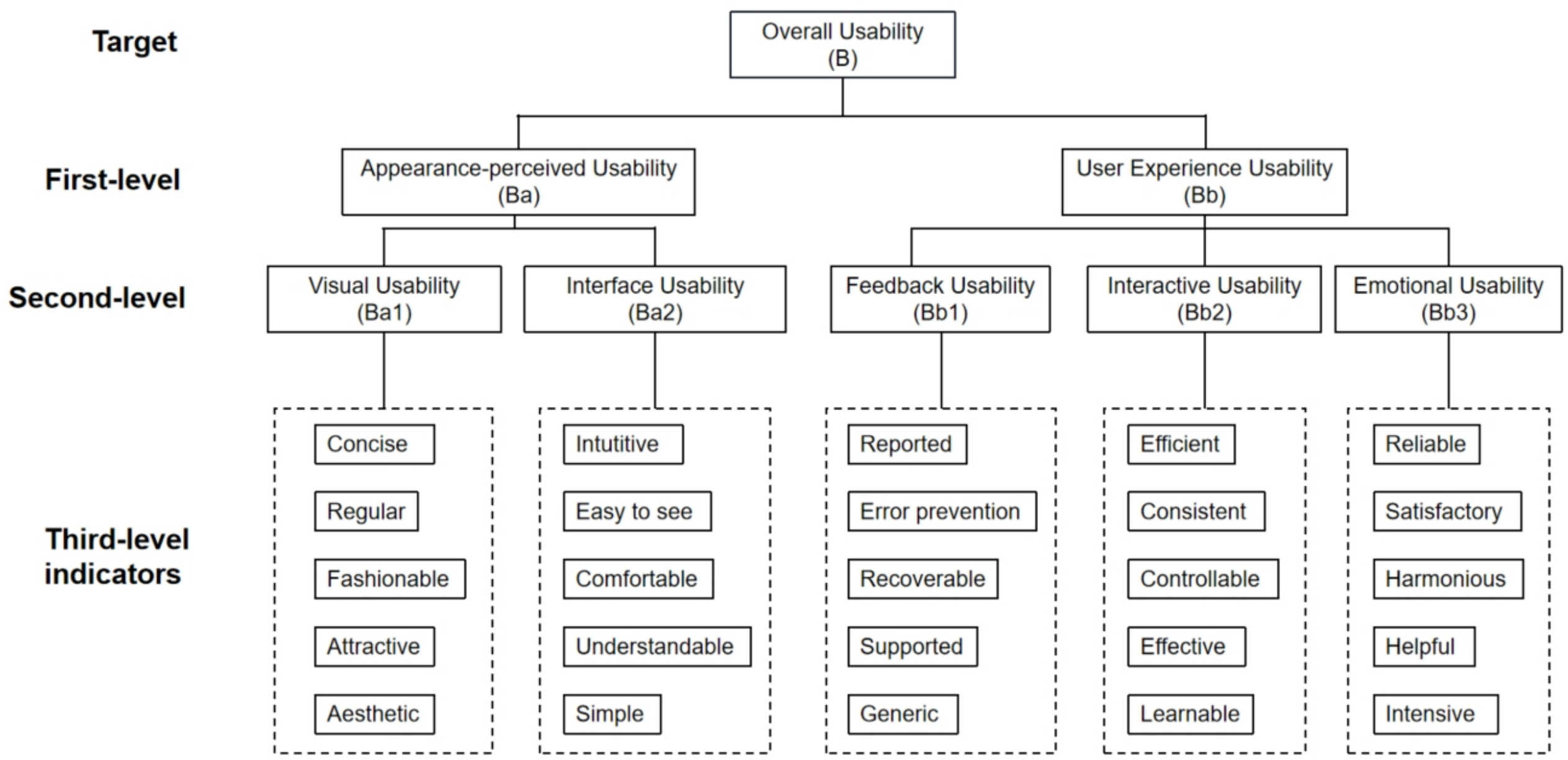

Supplementary Figure 4. Smart TV Usability Assessment Index System

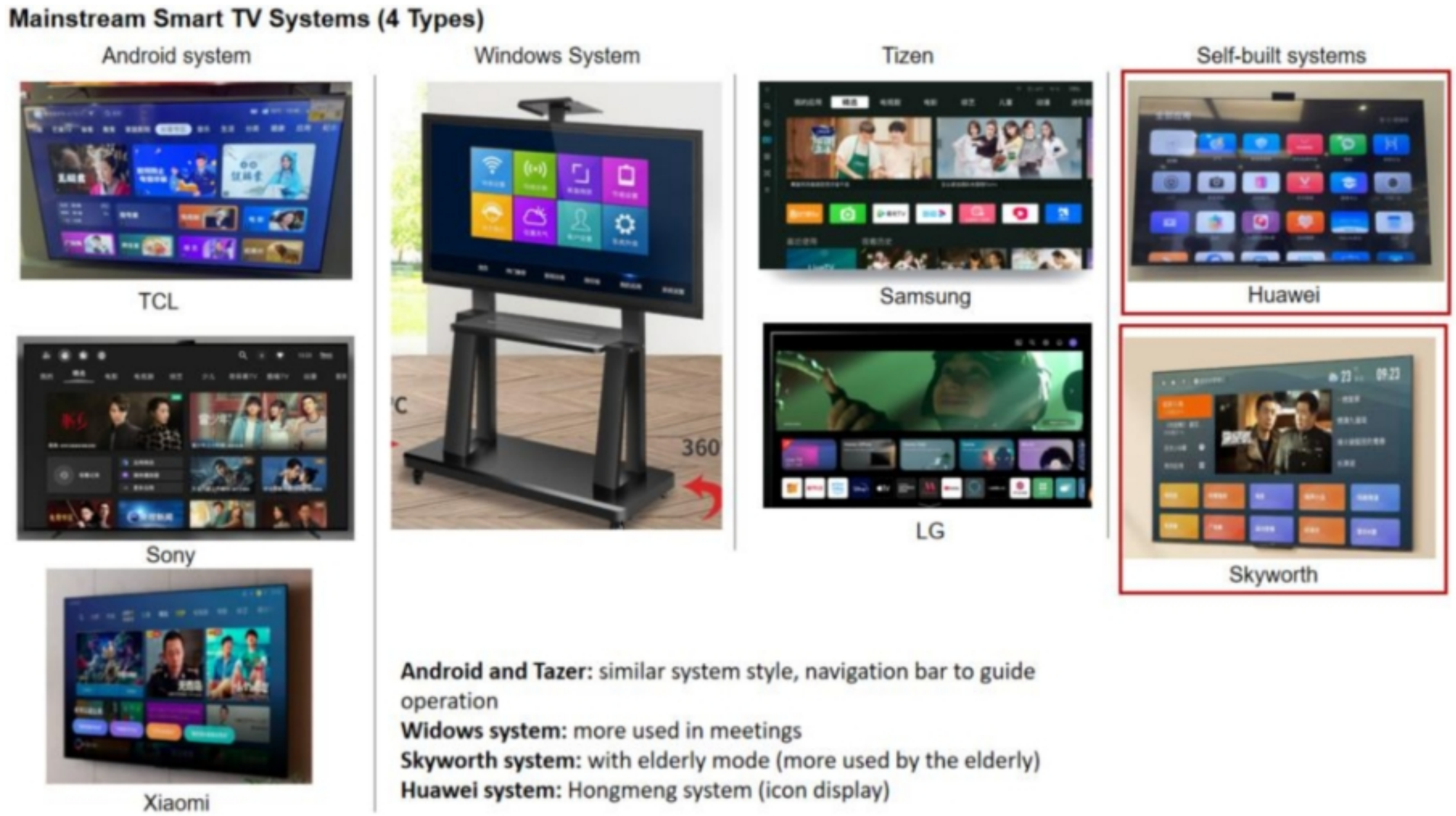

Supplementary Figure.5. Screening for Smart TVs samples

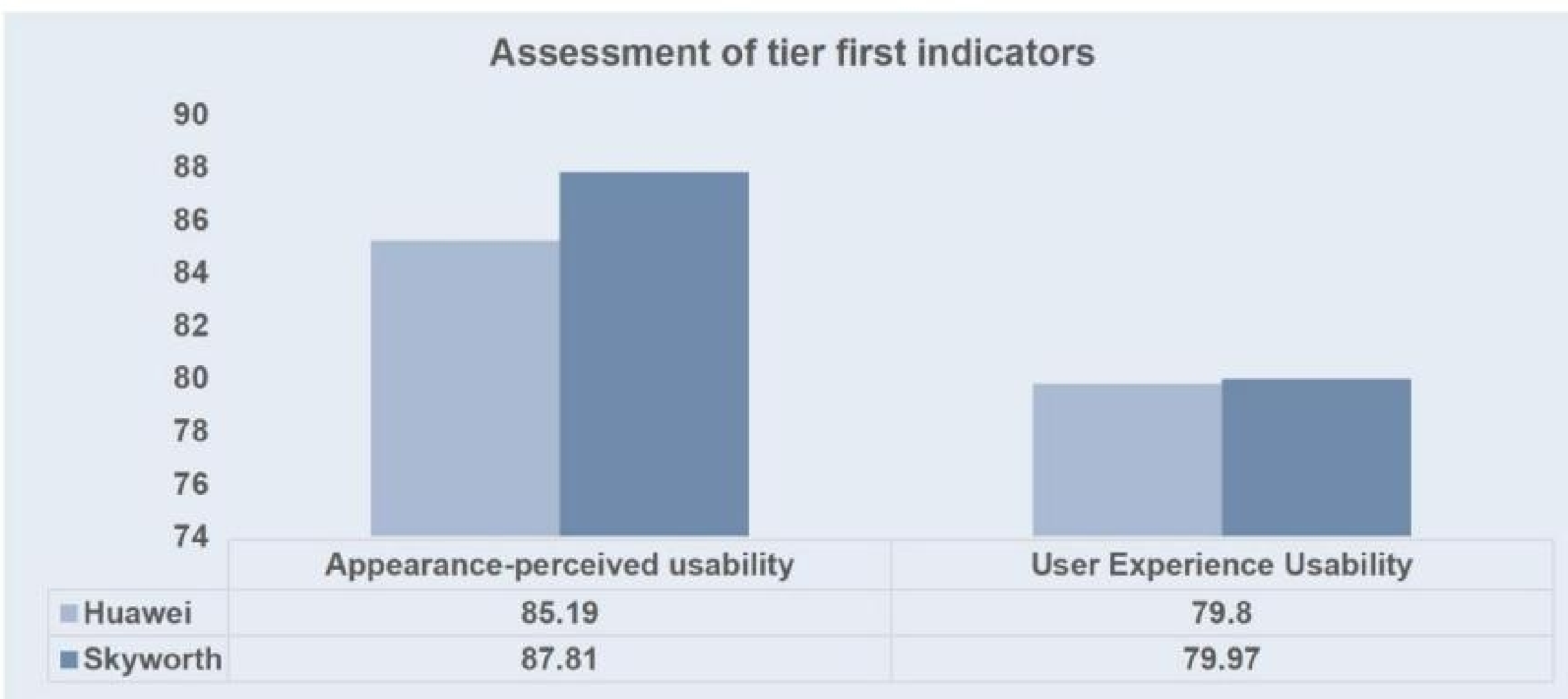

Supplementary Figure.6. Assessment of first-level Indicators

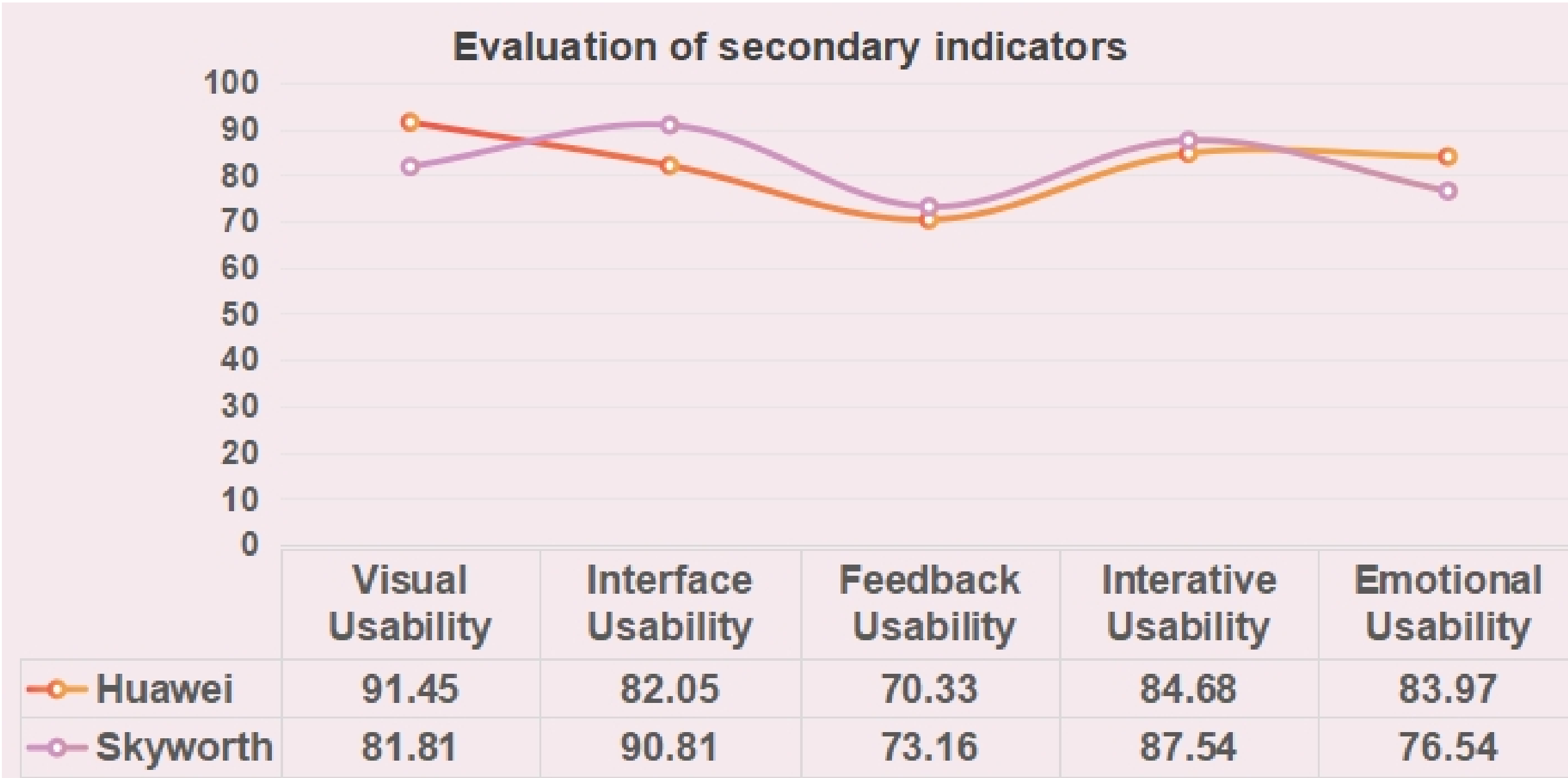

Supplementary Figure 7. Assessment of Second-level Indicators

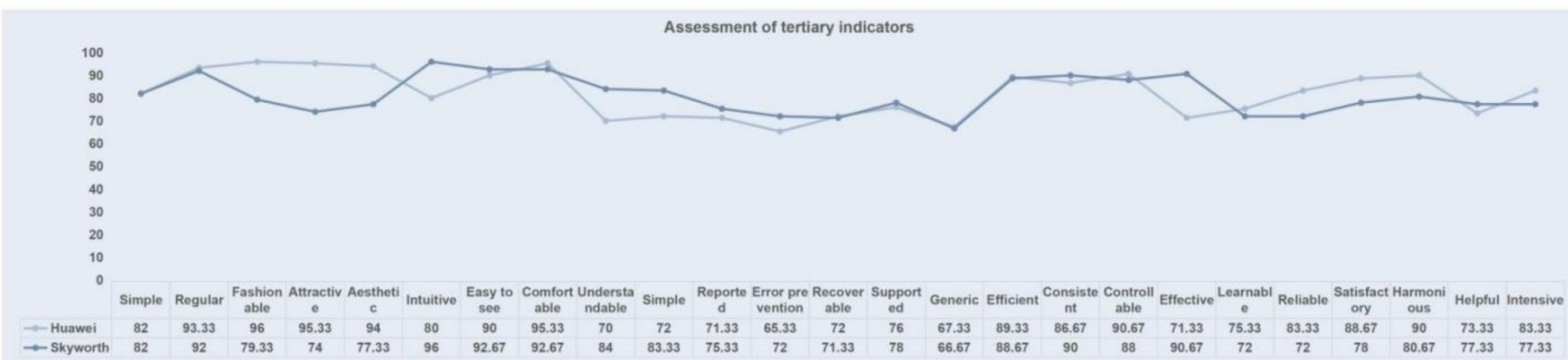

Supplementary Figure 8. Assessment of Third-level Indicators

1.2 Supplementary Tables

Supplementary Table 1. Background of Expert Validation

Expert	Category	Years of experience	Area of expertise
1	Innovative Design Services	5	Industrial Design
2	Information and Communication Technology.	7	Industrial Design
3	Aging Health Product Management	9	Industrial Design

Supplementary Table 2. Table of Meanings of the Values of the Comparison Matrix

Scale value	Meaning of materiality (ri/rj)
1	As important (rj or rj compared to self)
3	Slightly important
5	Important
7	Significantly important
9	Extremely important
2,4,6,8	Middle of the above values
The inverse of the above value	r_j/r_i

Supplementary Table 3. Target Layer: Overall Usability (B)

Overall Usability (B)	Appearance-perceived Usability (Ba)	User Experience Usability (Bb)	Local Weight
Ba	1	2/3	0.4000
Bb	3/2	1	0.6000

Maximum eigenvalue: λ_{max} = 2.0000

Consistency index: CI = 0.0000

Random consistency index: RI = 0.00

Consistency ratio: CR = 0.0000 < 0.1

Supplementary Table 4. First-level Dimension: Appearance-perceived Usability (Ba)

Appearance-perceived Usability (Ba)	Visual Usability (Ba1)	Interface Usability (Ba2)	Local Weight
Ba1	1	1/2	0.3333
Ba2	2	1	0.6667

λ_{max} = 2.0000, CI = 0.0000\), RI = 0.00, CR = 0.0000 < 0.1

Supplementary Table 5. First-level Dimension: User Experience Usability (Bb)

User Experience Usability (Bb)	Feedback Usability (Bb1)	Interactive Usability (Bb2)	Emotional Usability (Bb3)	Local Weight
Bb1	1	4/5	5/4	0.3275
Bb2	5/4	1	8/5	0.4126
Bb3	4/5	5/8	1	0.2599

λ_{max} = 3.0092, CI = 0.0046, RI = 0.58, CR = 0.0079 < 0.1

Supplementary Table 6. Second-level Dimension: Visual Usability (Ba1)

Visual Usability (Ba1)	Concise	Regular	Fashionable	Attractive	Aesthetic	Local Weight
Concise	1	1	1	2	2	0.2552
Regular	1	1	1	2	1	0.2222
Fashionable	1	1	1	3	1	0.2409
Attractive	1/2	1/2	1/3	1	2	0.1352
Aesthetic	1/2	1	1	1/2	1	0.1466

λ_{max} = 5.2850, CI = 0.0713, RI = 1.12, CR = 0.0636 < 0.1

Supplementary Table 7. Second-level Dimension: Interface Usability (Ba2)

Interface Usability (Ba2)	Intuitive	Easy to see	Comfortable	Understandable	Simple	Local Weight
Intuitive	1	1.5	1.5	1.8	2	0.2552
Easy to see	2/3	1	1	1.2	1.3	0.2222
Comfortable	2/3	1	1	1.2	1.3	0.2409
Understandable	5/9	5/6	5/6	1	1.1	0.1352
Simple	1/2	10/13	10/13	10/11	1	0.1466

λ_{max} ≈ 5.024, CI ≈ 0.006, RI = 1.12, CR ≈ 0.005 < 0.1

Supplementary Table 8. Second-level Dimension: Feedback Usability (Bb1)

Feedback Usability (Bb1)	Reported	Error prevention	Recoverable	Supported	Generic	Local Weight
Reported	1	1.5	1.8	2.3	2.6	0.3281
Error prevention	2/3	1	1.2	1.5	1.7	0.2164
Recoverable	5/9	5/6	1	1.3	1.5	0.1884
Supported	1/2.3	2/3	1/1.3	1	1.15	0.1428
Generic	1/2.6	1/1.7	2/3	1/1.15	1	0.1243

$\boldsymbol{\lambda_{\max} \approx 5.031, CI \approx 0.008, RI = 1.12, CR \approx 0.007 < 0.1}$

Supplementary Table 9. Second-level Dimension: Interactive Usability (Bb2)

Interactive Usability (Bb2)	Efficient	Consistent	Controllable	Effective	Learnable	Local Weight
Efficient	1	1.4	1.8	2	3.3	0.3232
Consistent	1/1.4	1	1.3	1.4	2.4	0.2312
Controllable	1/1.8	1/1.3	1	1.15	1.9	0.1856
Effective	1/1.2	1/1.4	1/1.15	1	1.6	0.1616
Learnable	1/3.3	1/2.4	1/1.9	1/1.6	1	0.0983

$\boldsymbol{\lambda_{\max} \approx 5.042, CI \approx 0.011, RI = 1.12, CR \approx 0.010 < 0.1}$

Supplementary Table 10. Second-level Dimension: Emotional Usability (Bb3)

Emotional Usability (Bb3)	Reliable	Satisfactory	Harmonious	Helpful	Intensive	Local Weight
Reliable	1	1.5	1.5	1.8	2	0.2947
Satisfactory	2/3	1	1	1.2	1.3	0.1944
Harmonious	2/3	1	1	1.2	1.3	0.1944
Helpful	5/9	5/6	5/6	1	1.1	0.1692
Intensive	1/2	10/13	10/13	10/11	1	0.1473

$\lambda_{\max} \approx 5.024$, $CI \approx 0.006$, $RI = 1.12$, $CR \approx 0.005 < 0.1$

Supplementary Table 11. Summary of Consistency Test for All Hierarchies

Hierarchy Level	Matrix Name	Order (n)	$\lambda_{\max}$	CI	RI	CR	Consistency
Target layer	Overall Usability	2	2.0000	0.0000	0.00	0.0000	Passed
First-level (Ba)	Appearance-perceived	2	2.0000	0.0000	0.00	0.0000	Passed
First-level (Bb)	User Experience	3	3.0092	0.0046	0.58	0.0079	Passed
Second-level (Ba1)	Visual Usability	5	5.2850	0.0713	1.12	0.0636	Passed
Second-level (Ba2)	Interface Usability	5	~5.024	~0.006	1.12	~0.005	Passed

Hierarchy Level	Matrix Name	Order (n)	$\lambda_{\max}$	CI	RI	CR	Consistency
Second-level (Bb1)	Feedback Usability	5	~5.031	~0.008	1.12	~0.007	Passed
Second-level (Bb2)	Interactive Usability	5	~5.042	~0.011	1.12	~0.010	Passed
Second-level (Bb3)	Emotional Usability	5	~5.024	~0.006	1.12	~0.005	Passed

Supplementary Table 12. Average Randomized Consistency Indicator Average Random Consistency Index (RI)

Ordinal Number	RI
1	0
2	0
3	0.52
4	0.89
5	1.12
6	1.26
7	1.36
8	1.41
9	1.46
10	1.49
11	1.52

Ordinal Number	RI
12	1.54
13	1.56
14	1.58
15	1.59

Supplementary Table 13. Intelligent Product Usability Weighting Values Results

Primary index	Weights	Secondary index	Weights	Tertiary index	Weights	Corresponding usability pain points of older adults
Appearance-perceived usability (Ba)	0.4000	Visual Usability (Ba1)	0.3333	Concise	0.2552	Reduced visual processing ability; difficulty in identifying cluttered interfaces
				Regular	0.2222	
				Fashionable	0.2409	
				Attractive	0.1352	
				Aesthetic	0.1466	
		Interface Usability (Ba2)	0.6667	Intuitive	0.2947	Declining working memory; difficulty in understanding complex navigation logic
				Easy to see	0.1944	
				Comfortable	0.1944	
				Understandable	0.1692	
				Simple	0.1473	
User Experience	0.6000	Feedback Usability (Bb1)	0.3275	Reported	0.3281	High perceptual threshold; difficulty in perceiving weak feedback; poor error recovery ability
				Error prevention	0.2164	

Usability (Bb)				Recoverable	0.1884	
				Supported	0.1428	
				Generic	0.1243	
		Interaction Usability (Bb2)	0.412 6	Efficient	0.3232	Slow motor response; high misoperation rate; difficulty in learning new operations
				Consistent	0.2312	
				Controllable	0.1856	
				Effective	0.1616	
				Learnable	0.0983	
		Emotional Usability (Bb3)	0.259 9	Reliable	0.2947	Operation anxiety; low confidence in using digital products
				Satisfactory	0.1944	
				Harmonious	0.1944	
				Helpful	0.1692	
				Intensive	0.1473	

Supplementary Table 14. Huawei vs Skyworth System Comparison

	**Huawei**	**Skyworth**
**Home Layout**	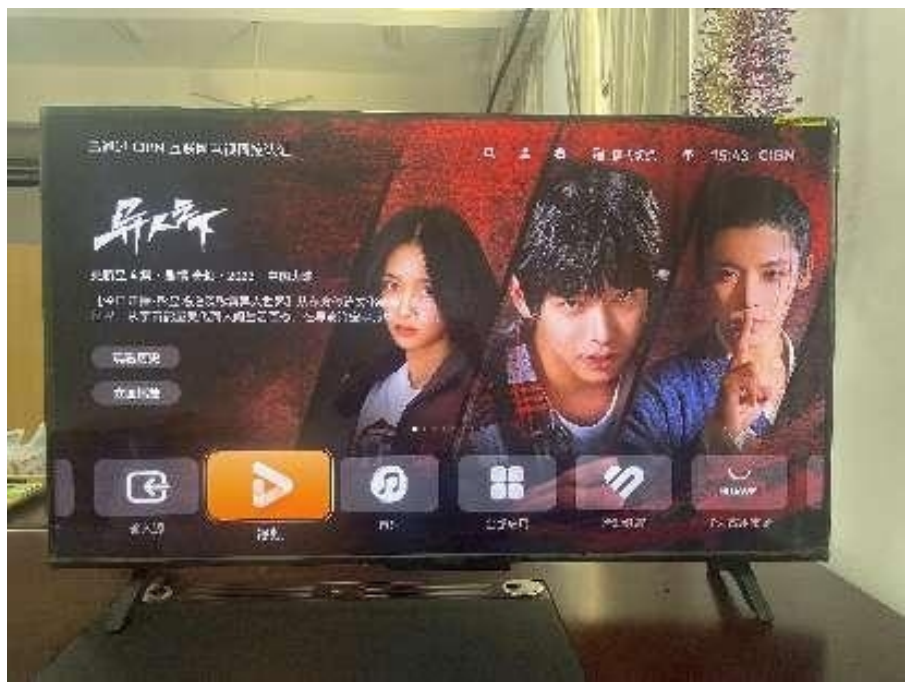	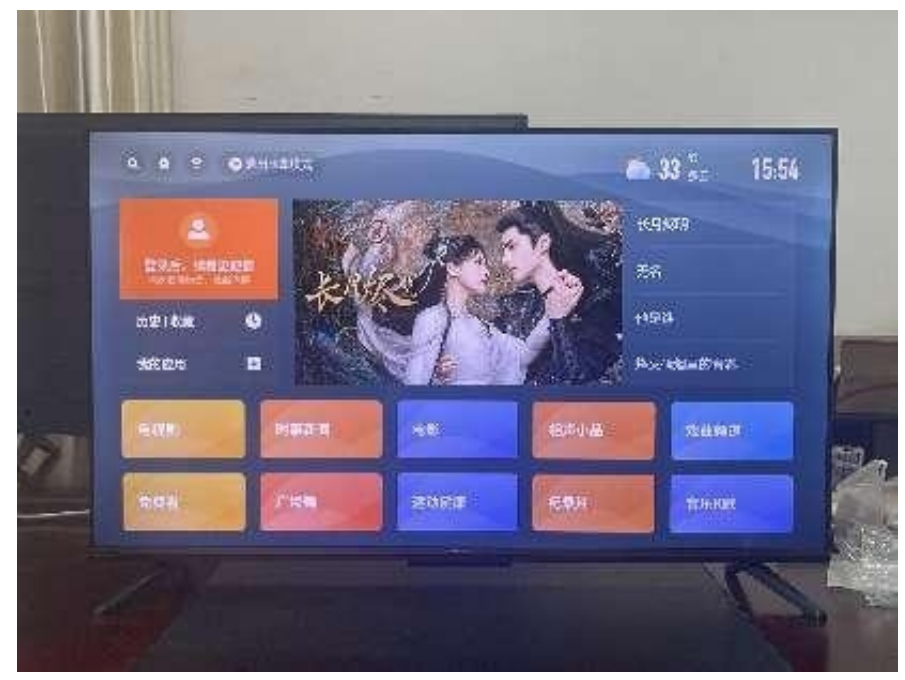
**Remote Control**	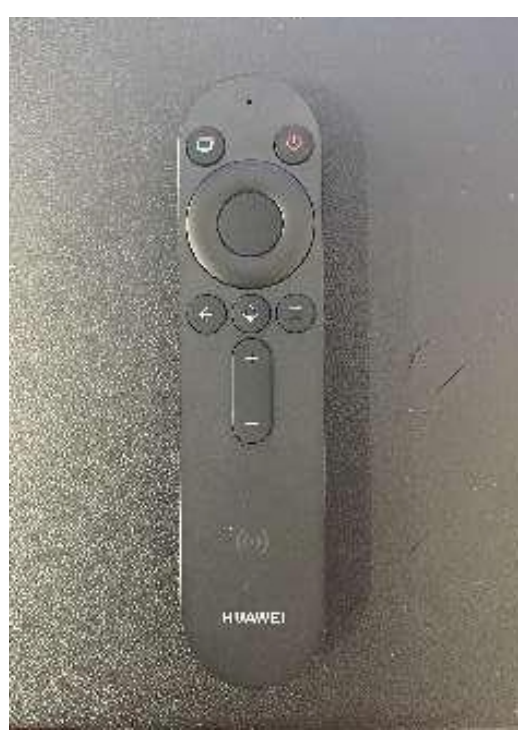	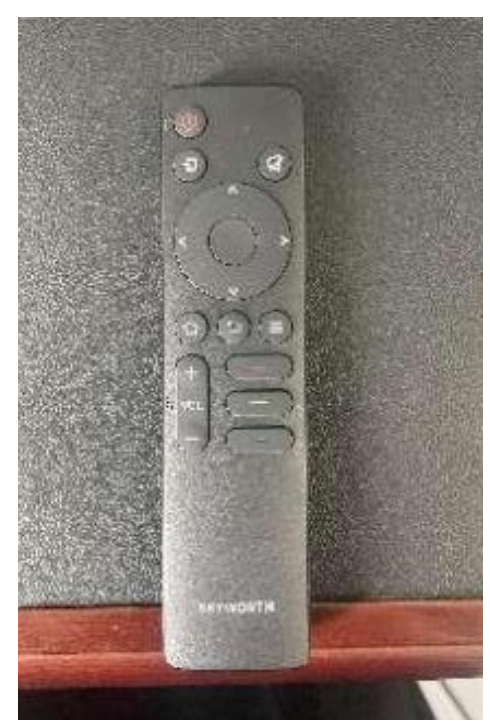

Supplementary Table 15. Tasks Flow

**Task Category**		
Search TV programs	Task 1	Search for a TV program via Pinyin input, play an episode, then return to the homepage.
	Task 2	Search for a TV program via voice input, play an episode, then return to the homepage
	Task 3	Search for a TV program via hierarchical navigation, play an episode, then return to the homepage.

Screen Mirroring	Task 4	Establish Bluetooth connection between TV and mobile phone for screen projection.
Video	Task 5	Project a mobile video to the TV, then return to the homepage.
Game	Task 6	Enter the game center, open a card game, play briefly, then return to the homepage.
Downloa d and uninstall	Task 7	Enter the app store, download and install an app, then uninstall it and return to the homepage.
Settings	Task 8	Enter settings, adjust image resolution to standard, then return to the homepage.

Supplementary Table 16. Demographic Background of 15 Older Users

Descriptions	Percentage (%)	Number (n)
(a) Gender<br>- Male<br>- Female	46<br>54	7<br>8
(b) Marital Status<br>- Married	100	15
(c) Educational Background<br>- Upper Secondary Education<br>-Tertiary education (college/university)	67<br>33	10<br>5
(d) Occupation<br>- Working<br>- Retired	14<br>86	2<br>13

Supplementary Table 17. Perturbation Scheme Design

Perturbation Type	Peturbation Range	Original Value	Peturbation Range	Adjusted Values
Weight perturbation	Feedback usability weight	0.3275	±5%, ±10%	+5%→0.3439; +10%→0.3603; -5%→0.3111; -10%→0.2948
Weight perturbation	Interaction usability weight	0.4126	±5%, ±10%	+5%→0.4332; +10%→0.4539; -5%→0.3920; -10%→0.3713
User rating perturbation	Error prevention (Huawei/Skyworth)	65.33/72.00	±1 point	Huawei: 64.33/66.33; Skyworth: 71.00/73.00
User rating perturbation	Recoverability (Huawei/Skyworth)	71.33/75.33	±1 point	Huawei: 70.33/72.33; Skyworth: 74.33/76.33

Supplementary Table 18. Weight Perturbation Sensitivity Analysis Results

Perturbed Parameter	Perturbation Range	Huawei's Score	Change Rate	Skyworth's Score	Change Rate
Feedback usability weight	+5%	82.40	+0.55%	83.58	+0.57%
	+10%	82.86	+1.11%	84.05	+1.13%
	-5%	81.50	-0.55%	82.64	-0.57%
	-10%	81.04	-1.11%	82.17	-1.13%
Interaction usability weight	+5%	82.28	+0.40%	83.82	+0.85%
	+10%	82.61	+0.80%	84.53	+1.71%
	-5%	81.62	-0.40%	82.40	-0.85%
	-10%	81.30	-0.80%	81.69	-1.71%

Supplementary Table 19. Impact of User Rating Perturbation on Feedback Usability Scores

Perturbed Indicator	Perturbation Range	Huawei's Feedback Usability Score	Change Rate	Skyworth's Feedback Usability Score	Change Rate
Error prevention	+1 point	70.88	+0.78%	73.71	+0.75%
	-1 point	69.78	-0.78%	72.61	-0.75%
Recoverability	+1 point	70.90	+0.81%	73.74	+0.80%
	-1 point	69.76	-0.81%	72.58	-0.80%
